# Illicit Drug Use, Illicit Drug Use Disorders, and Drug Overdose Deaths in Metropolitan and Nonmetropolitan Areas — United States

**DOI:** 10.15585/mmwr.ss6619a1

**Published:** 2017-10-20

**Authors:** Karin A. Mack, Christopher M. Jones, Michael F. Ballesteros

**Affiliations:** 1 National Center for Injury Prevention and Control, CDC; 2 Office of the Assistant Secretary for Planning and Evaluation, Office of the Secretary, U.S. Department of Health and Human Services

## Abstract

**Problem/Condition:**

Drug overdoses are a leading cause of injury death in the United States, resulting in approximately 52,000 deaths in 2015. Understanding differences in illicit drug use, illicit drug use disorders, and overall drug overdose deaths in metropolitan and nonmetropolitan areas is important for informing public health programs, interventions, and policies.

**Reporting Period:**

Illicit drug use and drug use disorders during 2003–2014, and drug overdose deaths during 1999–2015.

**Description of Data:**

The National Survey of Drug Use and Health (NSDUH) collects information through face-to-face household interviews about the use of illicit drugs, alcohol, and tobacco among the U.S. noninstitutionalized civilian population aged ≥12 years. Respondents include residents of households and noninstitutional group quarters (e.g., shelters, rooming houses, dormitories, migratory workers’ camps, and halfway houses) and civilians living on military bases. NSDUH variables include sex, age, race/ethnicity, residence (metropolitan/nonmetropolitan), annual household income, self-reported drug use, and drug use disorders.

National Vital Statistics System Mortality (NVSS-M) data for U.S. residents include information from death certificates filed in the 50 states and the District of Columbia. Cases were selected with an underlying cause of death based on the ICD-10 codes for drug overdoses (X40–X44, X60–X64, X85, and Y10–Y14). NVSS-M variables include decedent characteristics (sex, age, and race/ethnicity) and information on intent (unintentional, suicide, homicide, or undetermined), location of death (medical facility, in a home, or other [including nursing homes, hospices, unknown, and other locations]) and county of residence (metropolitan/nonmetropolitan).

Metropolitan/nonmetropolitan status is assigned independently in each data system. NSDUH uses a three-category system: Core Based Statistical Area (CBSA) of ≥1 million persons; CBSA of <1 million persons; and not a CBSA, which for simplicity were labeled large metropolitan, small metropolitan, and nonmetropolitan. Deaths from NVSS-M are categorized by the county of residence of the decedent using CDC’s National Center for Health Statistics 2013 Urban-Rural Classification Scheme, collapsed into two categories (metropolitan and nonmetropolitan).

**Results:**

Although both metropolitan and nonmetropolitan areas experienced significant increases from 2003–2005 to 2012–2014 in self-reported past-month use of illicit drugs, the prevalence was highest for the large metropolitan areas compared with small metropolitan or nonmetropolitan areas throughout the study period. Notably, past-month use of illicit drugs declined over the study period for the youngest respondents (aged 12–17 years). The prevalence of past-year illicit drug use disorders among persons using illicit drugs in the past year varied by metropolitan/nonmetropolitan status and changed over time. Across both metropolitan and nonmetropolitan areas, the prevalence of past-year illicit drug use disorders declined during 2003–2014.

In 2015, approximately six times as many drug overdose deaths occurred in metropolitan areas than occurred in nonmetropolitan areas (metropolitan: 45,059; nonmetropolitan: 7,345). Drug overdose death rates (per 100,000 population) for metropolitan areas were higher than in nonmetropolitan areas in 1999 (6.4 versus 4.0), however, the rates converged in 2004, and by 2015, the nonmetropolitan rate (17.0) was slightly higher than the metropolitan rate (16.2).

**Interpretation:**

Drug use and subsequent overdoses continue to be a critical and complicated public health challenge across metropolitan/nonmetropolitan areas. The decline in illicit drug use by youth and the lower prevalence of illicit drug use disorders in rural areas during 2012–2014 are encouraging signs. However, the increasing rate of drug overdose deaths in rural areas, which surpassed rates in urban areas, is cause for concern.

**Public Health Actions:**

Understanding the differences between metropolitan and nonmetropolitan areas in drug use, drug use disorders, and drug overdose deaths can help public health professionals to identify, monitor, and prioritize responses. Consideration of where persons live and where they die from overdose could enhance specific overdose prevention interventions, such as training on naloxone administration or rescue breathing. Educating prescribers on CDC’s guideline for prescribing opioids for chronic pain (Dowell D, Haegerich TM, Chou R. CDC guideline for prescribing opioids for chronic pain—United States, 2016. MMWR Recomm Rep 2016;66[No. RR-1]) and facilitating better access to medication-assisted treatment with methadone, buprenorphine, or naltrexone could benefit communities with high opioid use disorder rates.

## Introduction

During 1999–2014, annual age-adjusted death rates for the five leading causes of death in the United States (heart disease, cancer, unintentional injury, chronic lower respiratory disease, and stroke) were higher in rural (nonmetropolitan) areas than in urban (metropolitan) areas (*1*). Many factors influence the rural-urban mortality gap, including socioeconomic differences, health-related behaviors, and access to health care services. Residents of rural areas in the United States tend to be poorer and sicker than their urban counterparts, with rural residents in the South and West experiencing some of the most adverse health outcome (*2*).

In each year during 1999–2015, all-cause injury death rates were higher in nonmetropolitan areas of the United States than they were in metropolitan areas (*3*), and previous studies indicate that rates of drug overdose death and drug use varied markedly by metropolitan/nonmetropolitan status (*4*–*6*). Drug overdoses are now the leading cause of injury death in the United States, and although prescription drugs were primarily responsible for the rapid expansion of this large and growing public health crisis, illicit drugs (heroin, illicit fentanyl, cocaine, and methamphetamines) now are contributing substantially to the problem (*7*). Age-adjusted death rates for drug overdoses varied by the drug involved and the level of urbanization. For example, natural and semisynthetic opioid-related drug overdose death rates were highest and heroin-related drug overdose death rates were lowest in nonmetropolitan areas in 2015 compared with other levels of urbanicity (*7*).

A growing body of literature describes various aspects of the drug overdose epidemic and population density (*4*,*6*,*8*,*9*). Risk factors within metropolitan/nonmetropolitan status are a complicated mix of type of drug used (licit versus illicit), recreational versus pharmaceutical use, the combinations of drugs used, routes of administration (e.g., injection versus oral administration), the amount of drugs prescribed, the place used (home versus community), knowledge of potential adverse outcomes, access to overdose reversal drugs, and access to emergency services and substance abuse treatment services.

This report examines trends in drug use, drug use disorders, and overdose deaths in metropolitan and nonmetropolitan areas of the United States through analyses of 2003–2014 National Survey of Drug Use and Health (NSDUH) data and 1999–2015 National Vital Statistics System Mortality (NVSS-M) data. Public health professionals and clinicians can use these findings to identify specific subgroups and rural/urban groups in need of targeted interventions.

## Methods

### Data Sources

Drug use and drug use disorder data were from the 2003–2014 NSDUH, which is managed by the Substance Abuse and Mental Health Services Administration’s Center for Behavioral Health Statistics and Quality (https://www.samhsa.gov/data/population-data-nsduh). NSDUH collects information through face-to-face household interviews about the use of illicit drugs, alcohol, and tobacco among the U.S. noninstitutionalized civilian population aged ≥12 years. Respondents include residents of households and noninstitutional group quarters (e.g., shelters, rooming houses, dormitories, migratory workers’ camps, and halfway houses) and civilians living on military bases. An independent, multistage area probability sample design for each of the 50 states and the District of Columbia allows for the production of state-level and urban status (county of residence) estimates.

Mortality data for U.S. residents were from the 1999–2015 NVSS-M, which is based on information from all death certificates filed in the 50 states and the District of Columbia. Deaths of nonresidents (e.g., nonresident aliens; nationals living abroad; and residents of Puerto Rico, Guam, the U.S. Virgin Islands, and other U.S. territories) were excluded. Mortality data are provided to CDC’s National Center for Health Statistics (NCHS) through the Vital Statistics Cooperative Program and coded according to the International Classification of Diseases, 10th Revision (ICD-10) by NCHS. Analyses were restricted to deaths with an underlying cause of death based on the ICD-10 codes for drug overdoses (X40–X44, X60–X64, X85, and Y10–Y14) (*10*).

### Variables

NSDUH variables included sex, age, race/ethnicity, residence (metropolitan/nonmetropolitan area), annual household income, self-reported drug use, and drug use disorders. Metropolitan/nonmetropolitan status was coded using NSDUH’s Core Based Statistical Area (CBSA) measure, which was available across the study period. This measure uses three segments of population density: CBSA with population of ≥1 million persons (large metropolitan); CBSA with population of <1 million persons (small metropolitan); and not a CBSA (nonmetropolitan) (*11*,*12*). For this report, CBSAs with populations ≥1 million persons and <1 million persons were considered metropolitan and those classed as “not a CBSA” were considered nonmetropolitan.

Self-reported drug use in NSDUH included past-month use of illicit drugs (marijuana/hashish, cocaine [including crack], inhalants, hallucinogens, or heroin0 or nonmedical use of prescription-type drugs (opioids, sedatives, tranquilizers, stimulants). The presence of a past-year illicit drug use disorder was defined using criteria specified within the 4th edition of the Diagnostic and Statistical Manual of Mental Disorders, which include symptoms such as withdrawal, tolerance, use in dangerous situations, trouble with the law, and interference with major obligations at work, school, or home (*13*). Respondents were asked questions about substance use disorders if they had reported use of illicit drugs in the past 12 months. The full survey instrument is available at http://www.icpsr.umich.edu/icpsrweb/NAHDAP/studies/36361.

NVSS-M variables included decedent characteristics (sex, age, and race/ethnicity) and information on the intent (unintentional, suicide, homicide, or undetermined) and location of death (medical facility, in a home, or other [including nursing homes, hospices, unknown, and other locations], and county of residence [metropolitan/nonmetropolitan area]). Location of death might be different from location of drug use. NVSS-M suppression rules include not reporting cell counts with <10 persons and rates are considered unreliable for <20 deaths. Deaths were categorized as metropolitan or nonmetropolitan based on the county of residence. Nonmetropolitan and metropolitan areas were identified using the NCHS 2013 county-based classification scheme (*14*). The six NCHS classification levels for counties are: 1) large central metropolitan: part of a metropolitan statistical area with ≥1 million population and covers a principal city; 2) large fringe metropolitan: part of a metropolitan statistical area with ≥1 million population but does not cover a principal city; 3) medium metropolitan: part of a metropolitan statistical area with ≥250,000 but <1 million population; 4) small metropolitan: part of a metropolitan statistical area with <250,000 population; 5) micropolitan (nonmetropolitan): part of a micropolitan statistical area (has an urban cluster of ≥10,000 but <50,000 population); and 6) noncore (nonmetropolitan): not part of a metropolitan or micropolitan statistical area. For a dichotomous measure, metropolitan (urban) combines categories 1–4 and nonmetropolitan (rural) combines 5 and 6.

### Data Analysis

On the basis of NSDUH data, overall prevalence of past-month illicit drug use was estimated for four 3-year periods (2003–2005, 2006–2008, 2009–2011, and 2012–2014), by CBSA designation, sex, age group, race, and annual household income. Prevalence of past-year illicit drug use disorder among persons reporting past-year illicit drug use was calculated by large metropolitan, small metropolitan and nonmetropolitan areas for the four periods, overall, and by sex. Estimates were weighted based on the complex sample design and sampling weights of the NSDUH. Years were pooled to improve the precision of estimates and enable comparisons across subgroups. Logistic regression models (presence of drug use or drug use disorder) tested p-values of beta coefficients of the year variable to assess statistically significant (p <0.05) changes in trends. Percentage change was calculated by comparing the early period (2003–2005) with the last period (2012–2014). The analysis of trends in age-adjusted death rates during 1999–2015 included all ages; death rates per 100,000 persons were adjusted to the 2000 U.S. standard population by the direct method (*10*).

## Results

### National Survey of Drug Use and Health

From 2003–2005 to 2012–2014, the prevalence of past-month use of illicit drugs was highest in large metropolitan areas (Table 1). All three urban status groups (large metropolitan, small metropolitan, and nonmetropolitan) experienced significant increases in the prevalence of past-month drug use overall. Prevalence was higher for males than females during all time intervals in all urban status groups. However, in the large metropolitan group, the percentage increase in prevalence from 2003–2005 to 2012–2014 was greater for females (23.4%) than for males (21.6%). The prevalence of illicit drug use among nonmetropolitan females remained stable during the study period.

**TABLE 1:** Prevalence of self-reported past-month use of illicit drugs, by metropolitan/nonmetropolitan areas,* sex, age, race, and annual household income — National Survey of Drug Use and Health, United States, 2003–2014

Characteristic	% of respondents reporting use				% change 2003–2005 to 2012–2014	Trend (p value^†^)
	2003–2005	2006–2008	2009–2011	2012–2014		
**Overall**						
Large metropolitan	8.3	8.5	9.3	10.1	21.7	<0.001
Small metropolitan	8.2	7.9	8.7	9.5	15.9	<0.001
Nonmetropolitan	6.0	5.9	6.6	6.8	13.3	0.036
**Sex**						
Females						
Large metropolitan	6.4	6.3	7.1	7.9	23.4	<0.001
Small metropolitan	6.4	5.9	6.5	6.9	7.8	0.005
Nonmetropolitan	4.6	4.7	4.8	4.7	2.2	0.856
Males						
Large metropolitan	10.2	10.9	11.6	12.4	21.6	<0.001
Small metropolitan	10.3	10.0	10.9	12.2	18.4	<0.001
Nonmetropolitan	7.5	7.2	8.5	8.9	18.7	0.029
**Age group (yrs)**						
12–17						
Large metropolitan	10.4	9.7	10.4	9.3	-10.6	0.029
Small metropolitan	11.2	9.4	10.3	9.4	-16.1	<0.001
Nonmetropolitan	9.1	7.5	8.3	7.9	-13.2	0.271
18–25						
Large metropolitan	20.3	20.4	22.2	23.1	13.8	<0.001
Small metropolitan	20.5	20.0	21.6	20.5	0.0	0.354
Nonmetropolitan	16.0	15.7	16.1	15.6	-2.5	0.840
26–34						
Large metropolitan	11.6	11.6	13.9	15.3	31.9	<0.001
Small metropolitan	10.8	11.0	11.9	14.3	32.4	<0.001
Nonmetropolitan	9.4	8.9	12.1	12.2	29.8	0.024
≥35						
Large metropolitan	4.5	5.0	5.2	6.1	35.6	<0.001
Small metropolitan	4.3	4.4	4.9	6.1	41.9	<0.001
Nonmetropolitan	3.3	3.7	3.9	4.5	36.4	0.036
**Race/Ethnicity**						
Non-Hispanic white						
Large metropolitan	8.8	9.1	9.9	10.9	23.9	<0.001
Small metropolitan	8.1	8.1	7.9	8.7	7.4	<0.001
Nonmetropolitan	5.6	5.6	6.0	6.3	12.5	0.133
Other						
Large metropolitan	7.4	7.7	8.4	9.1	23.0	<0.001
Small metropolitan	8.6	8.0	8.6	9.8	14.0	<0.001
Nonmetropolitan	8.3	7.4	9.9	9.0	8.4	0.368
**Annual household income**						
<$20,000						
Large metropolitan	10.1	11.5	12.9	13.9	37.6	<0.001
Small metropolitan	11.8	11.4	13.7	14.8	25.4	<0.001
Nonmetropolitan	8.5	8.4	9.2	9.9	16.5	0.158
$20,000–$49,999						
Large metropolitan	8.9	9.4	9.2	10.8	21.3	<0.001
Small metropolitan	8.1	8.1	9.2	9.8	21.0	<0.001
Nonmetropolitan	5.7	5.7	6.7	7.4	29.8	0.011
$50,000–$74,999						
Large metropolitan	8.1	8.1	8.8	9.7	19.8	0.001
Small metropolitan	6.8	6.7	6.5	7.2	5.9	0.479
Nonmetropolitan	4.5	5.0	5.3	4.5	0.0	0.910
≥$75,000						
Large metropolitan	6.6	6.7	7.9	8.1	22.7	0.001
Small metropolitan	6.0	6.0	5.8	6.8	13.3	0.075
Nonmetropolitan	4.1	3.9	4.3	3.6	-12.2	0.671

* Based on the National Survey of Drug Use and Health’s measure of population density: core based statistical area (CBSA) of ≥1 million persons = large metropolitan area; CBSA of <1 million persons = small metropolitan area; and not a CBSA = nonmetropolitan. The full survey instrument is available at http://www.icpsr.umich.edu/icpsrweb/NAHDAP/studies/36361.

^†^ P value determined by bivariate logistic regression.

During 2012–2014, respondents aged 18–25 years had the highest prevalence of past-month use of illicit drugs for all urban levels (Table 1). For respondents in this age group, the prevalence increased slightly from 2003–2005 to 2012–2014 in large metropolitan areas (13.8%) while the prevalence remained stable among small metropolitan area respondents and nonmetropolitan respondents. Past-month use of illicit drugs declined over the study period for the youngest respondents (aged 12–17 years), with the largest decline among small metropolitan area youth. Past-month use of illicit drugs increased significantly among all three urban areas among persons aged 26–34 years and those aged ≥35 years.

Prevalence of past-month illicit drug use increased among both non-Hispanic whites and other races for both large and small metropolitan areas (Table 1). Prevalence did not change among non-Hispanic white nonmetropolitan respondents nor among nonmetropolitan respondents of other races. When past-month illicit drug use was examined by annual household income, respondents with an annual household income <$20,000 had the highest prevalence across all three geographic groups. Persons living in large metropolitan areas with a household income of <$20,000 experienced the largest increase in past-month illicit drug use during the study period (37.6%), followed by nonmetropolitan residents with a household income of $20,000–$49,999 (29.8%).

**FIGURE 1:**
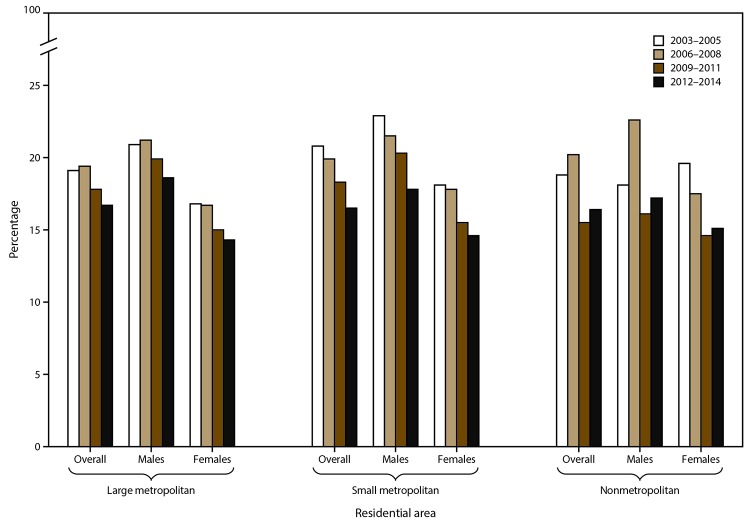
Trends* in prevalence of past-year illicit drug use disorder among persons reporting past-year illicit drug use, by sex and residential area^†^ — National Survey of Drug Use and Health, United States, 2003–2014 * Bivariate logistic regression models were used to test statistically significant changes in trends between 2003–2005 and 2012–2014; p<0.05 was the measure of significance in each case, except for nonmetropolitan females, for whom the trend was not significant. **^†^** Based on the National Survey of Drug Use and Health’s measure of population density: core based statistical area (CBSA) of ≥1 million persons = large metropolitan area; CBSA of <1 million persons = small metropolitan area; and not a CBSA = nonmetropolitan. The full survey instrument is available at http://www.icpsr.umich.edu/icpsrweb/NAHDAP/studies/36361.

The prevalence of past-year illicit drug use disorders among persons reporting illicit drug use in the past year varied by metropolitan/nonmetropolitan area and sex, and changed over time (Figure 1). All three geographic groups experienced statistically significant declines in overall prevalence of drug use disorders during the study period. For residents in large metropolitan areas, prevalence declined 12.6%. For residents in small metropolitan areas, prevalence declined 20.7% from 20.8% during 2003–2005 to 16.5% during 2012–2014. Among nonmetropolitan residents, the prevalence of past-year illicit drug use disorders decreased 12.8%, from 18.8% during 2003–2005 to 16.4% during 2012–2014. During 2012–2014, prevalence rates were similar across the three geographic groups.

Males who reported illicit drug use in the past year consistently had higher prevalence of illicit drug use disorders compared with females. In general, females experienced consistently larger declines during the study period. The prevalence of illicit drug use disorders among females declined 14.9% among those living in large metropolitan areas, 19.3% among those living in small metropolitan areas, and 23.0% among nonmetropolitan residents. The prevalence of illicit drug use disorders declined significantly in metropolitan areas for males and females. The decline in prevalence of illicit drug use disorders among nonmetropolitan residents was significant overall and for males during the study period.

### National Vital Statistics System

In 2015, nearly six times as many drug overdose deaths were reported in metropolitan areas than in nonmetropolitan areas (metropolitan: 45,059; nonmetropolitan: 7,345) in the United States (Table 2). The overall percentage change in the number of deaths for nonmetropolitan areas over the time period was 325% (the percentage change in the age-adjusted death rate per 100,000 was 330%; data not reported). Although age-adjusted drug overdose death rates for metropolitan areas were higher than in nonmetropolitan areas in 1999 (6.4 versus 4.0, respectively), the rates converged in 2004 (9.4 in both areas), and in 2015 the nonmetropolitan rate (17.0) was slightly higher than the metropolitan rate (16.2).

**TABLE 2:** Number and age-adjusted rate per 100,000 persons for self-reported drug overdose deaths, by sex, race/ethnicity, intent of death, and age-group, for metropolitan and nonmetropolitan counties of residence — National Vital Statistics System, United States, 1999–2015*

Characteristic																			
Urbanicity	No. of deaths (age-adjusted rate)																		
		1999	2000	2001	2002	2003	2004	2005	2006	2007	2008	2009	2010	2011	2012	2013	2014	2015	% change 1999/2015
Metro		15,120 (6.4)	15,408 (6.5)	16,937 (7.0)	20,512 (8.4)	22,263 (9.0)	23,394 (9.4)	25,520 (10.1)	29,321 (11.5)	30,604 (11.8)	30,862 (11.8)	31,266 (11.8)	32,323 (12.1)	34,853 (12.9)	35,264 (13.0)	37,547 (13.7)	40,272 (14.6)	45,059 (16.2)	198
Nonmetro		1,729 (4.0)	2,007 (4.6)	2,457 (5.7)	3,006 (6.9)	3,522 (8.2)	4,030 (9.4)	4,293 (9.9)	5,104 (11.7)	5,406 (12.3)	5,588 (12.7)	5,738 (12.9)	6,006 (13.6)	6,487 (14.7)	6,238 (14.2)	6,435 (14.6)	6,783 (15.6)	7,345 (17.0)	325
All deaths		16,849 (6.1)	17,415 (6.2)	19,394 (6.8)	23,518 (8.2)	25,785 (8.9)	27,424 (9.4)	29,813 (10.1)	34,425 (11.5)	36,010 (11.9)	36,450 (11.9)	37,004 (11.9)	38,329 (12.3)	41,340 (13.2)	41,502 (13.1)	43,982 (13.8)	47,055 (14.7)	52,404 (16.3)	211
**Sex**																			
Metro females		4,895 (4.1)	5,058 (4.2)	5,778 (4.7)	7,322 (5.9)	8,018 (6.4)	8,705 (6.8)	9,363 (7.3)	10,520 (8.1)	11,486 (8.7)	11,643 (8.7)	11,926 (8.8)	12,795 (9.4)	13,572 (9.9)	13,672 (9.8)	14,403 (10.3)	15,290 (10.8)	16,321 (11.5)	233
Nonmetro females		696 (3.1)	794 (3.6)	958 (4.3)	1,168 (5.4)	1,368 (6.3)	1,599 (7.3)	1,726 (7.9)	2,012 (9.1)	2,226 (10.0)	2,339 (10.5)	2,485 (11.1)	2,528 (11.5)	2,780 (12.5)	2,718 (12.4)	2,780 (12.6)	2,953 (13.4)	3,126 (14.3)	349
Metro males		10,225 (8.8)	10,350 (8.8)	11,159 (9.4)	13,190 (11.0)	14,245 (11.7)	14,689 (12.0)	16,157 (13.0)	18,801 (14.9)	19,118 (15.0)	19,219 (14.9)	19,340 (14.9)	19,528 (14.9)	21,281 (16.1)	21,592 (16.2)	23,144 (17.2)	24,982 (18.4)	28,738 (21.0)	181
Nonmetro males		1,033 (4.8)	1,213 (5.6)	1,499 (6.9)	1,838 (8.5)	2,154 (9.9)	2,431 (11.3)	2,567 (11.8)	3,092 (14.1)	3,180 (14.5)	3,249 (14.7)	3,253 (14.6)	3,478 (15.6)	3,707 (16.7)	3,520 (16.0)	3,655 (16.6)	3,830 (17.6)	4,219 (19.5)	308
**Race/Ethnicity^†^**																			
Metro AI/AN		100 (7.1)	79 (5.5)	106 (7.4)	134 (9.1)	172 (11.9)	200 (13.6)	185 (12.7)	211 (14.1)	217 (14.4)	236 (15.2)	264 (17.3)	255 (16.5)	276 (18.0)	301 (18.9)	304 (18.8)	343 (21.4)	361 (22.1)	261
Nonmetro AI/AN		31 (3.9)	47 (5.5)	51 (6.1)	66 (7.5)	77 (8.9)	94 (10.6)	125 (13.9)	126 (14.0)	128 (14.1)	157 (17.0)	169 (18.2)	165 (17.3)	177 (18.5)	181 (18.8)	173 (18.5)	191 (20.2)	192 (19.8)	519
Metro black		2,321 (8.0)	2,305 (7.9)	2,422 (8.1)	2,688 (8.8)	2,723 (8.8)	2,762 (8.8)	3,147 (9.8)	3,715 (11.4)	3,407 (10.3)	2,993 (8.9)	2,983 (8.6)	2,950 (8.4)	3,195 (9.0)	3,329 (9.2)	3,723 (10.1)	4,124 (11.1)	4,802 (12.7)	107
Nonmetro black		108 (3.1)	86 (2.5)	97 (2.7)	96 (2.6)	99 (2.8)	133 (3.6)	175 (4.8)	197 (5.4)	167 (4.5)	154 (4.2)	177 (4.7)	172 (4.5)	190 (5.0)	159 (4.2)	205 (5.3)	199 (5.3)	268 (7.1)	148
Metro Hispanic^§^		1,581 (5.5)	1,356 (4.6)	1,401 (4.5)	1,719 (5.4)	1,888 (5.7)	1,786 (5.2)	2,079 (5.8)	2,352 (6.3)	2,325 (5.9)	2,342 (5.8)	2,418 (5.8)	2,375 (5.6)	2,715 (6.1)	2,832 (6.2)	3,110 (6.6)	3,246 (6.7)	3,852 (7.7)	144
Nonmetro Hispanic		88 (4.5)	87 (4.3)	82 (3.7)	112 (4.9)	121 (5.0)	126 (5.2)	141 (5.4)	167 (6.3)	162 (5.6)	194 (6.8)	183 (6.1)	208 (6.8)	226 (7.2)	223 (7.3)	235 (7.4)	258 (7.7)	265 (7.5)	201
Metro A/PI		118 (1.1)	114 (1.0)	140 (1.2)	168 (1.4)	181 (1.4)	191 (1.5)	214 (1.6)	261 (1.9)	263 (1.8)	246 (1.6)	297 (1.9)	309 (1.9)	387 (2.3)	389 (2.2)	407 (2.3)	449 (2.4)	530 (2.7)	349
Nonmetro A/PI		11 (U)	11 (U)	—**^¶^**	—	10 (U)	—	20 (4.6)	—	14 (U)	22 (4.5)	12 (U)	13 (U)	15 (U)	14 (U)	17 (U)	16 (U)	18 (U)	64
Metro white		10,844 (6.6)	11,402 (6.9)	12,727 (7.7)	15,688 (9.5)	17,172 (10.5)	18,369 (11.2)	19,780 (12.0)	22,678 (13.7)	24,293 (14.7)	24,913 (15.0)	25,120 (15.1)	26,280 (15.8)	28,098 (17.0)	28,178 (17.0)	29,788 (17.9)	31,853 (19.3)	35,148 (21.4)	224
Nonmetro white		1,484 (4.0)	1,769 (4.8)	2,222 (6.2)	2,718 (7.6)	3,203 (9.0)	3,655 (10.4)	3,824 (10.8)	4,596 (13.0)	4,924 (13.9)	5,053 (14.2)	5,183 (14.5)	5,431 (15.3)	5,863 (16.6)	5,645 (16.1)	5,793 (16.5)	6,092 (17.6)	6,572 (19.2)	343
**Intent of death**																			
Metro unintentional		10,058 (4.3)	10,429 (4.4)	11,364 (4.7)	14,308 (5.9)	15,678 (6.4)	16,868 (6.8)	19,213 (7.6)	22,533 (8.9)	23,491 (9.1)	23,844 (9.1)	24,248 (9.2)	25,256 (9.5)	27,845 (10.4)	28,147 (10.4)	30,534 (11.2)	33,155 (12.1)	38,039 (13.8)	278
Nonmetro unintentional		1,097 (2.5)	1,283 (2.9)	1,660 (3.8)	2,086 (4.8)	2,616 (6.1)	2,970 (6.9)	3,235 (7.5)	3,867 (8.9)	4,167 (9.6)	4,327 (9.9)	4,506 (10.2)	4,750 (10.9)	5,226 (11.9)	5,028 (11.6)	5,129 (11.8)	5,563 (12.9)	6,087 (14.2)	455
Metro suicide		2,767 (1.2)	2,751 (1.2)	3,042 (1.3)	3,291 (1.4)	3,338 (1.3)	3,526 (1.4)	3,613 (1.4)	3,854 (1.5)	4,076 (1.5)	4,310 (1.6)	4,336 (1.6)	4,535 (1.7)	4,552 (1.6)	4,719 (1.7)	4,650 (1.7)	4,663 (1.6)	4,445 (1.5)	61
Nonmetro suicide		414 (0.9)	492 (1.1)	517 (1.2)	593 (1.3)	552 (1.3)	682 (1.5)	627 (1.4)	717 (1.6)	696 (1.5)	717 (1.6)	705 (1.5)	763 (1.6)	746 (1.6)	746 (1.6)	782 (1.7)	770 (1.6)	761 (1.6)	84
Metro undetermined		2,264 (1.0)	2,199 (0.9)	2,494 (1.0)	2,876 (1.2)	3,205 (1.3)	2,941 (1.2)	2,647 (1.1)	2,874 (1.1)	2,989 (1.2)	2,654 (1.0)	2,625 (1.0)	2,484 (0.9)	2,399 (0.9)	2,333 (0.8)	2,306 (0.8)	2,397 (0.9)	2,509 (0.9)	11
Nonmetro undetermined		209 (0.5)	230 (0.5)	275 (0.6)	321 (0.7)	348 (0.8)	365 (0.8)	421 (1.0)	505 (1.2)	530 (1.2)	530 (1.2)	514 (1.1)	479 (1.1)	492 (1.1)	449 (1.0)	495 (1.1)	426 (1.0)	470 (1.1)	125
Metro homicide		31 (0.0)	29 (0.0)	37 (0.0)	37 (0.0)	42 (0.0)	59 (0.0)	47 (0.0)	60 (0.0)	48 (0.0)	54 (0.0)	57 (0.0)	48 (0.0)	57 (0.0)	65 (0.0)	57 (0.0)	57 (0.0)	66 (0.0)	113
Nonmetro homicide		—	—	—	—	—	13 (U)	10 (U)	15 (U)	13 (U)	14 (U)	13 (U)	14 (U)	23 (0.1)	15 (U)	29 (0.1)	24 (0.0)	27 (0.1)	200
Age group (yrs)**																			
Metro 0–11		43 (0.1)	36 (0.1)	48 (0.1)	57 (0.1)	60 (0.1)	63 (0.2)	67 (0.2)	80 (0.2)	72 (0.2)	79 (0.2)	68 (0.2)	68 (0.2)	68 (0.2)	75 (0.2)	64 (0.2)	63 (0.2)	84 (0.2)	95
Nonmetro 0–11		14 (U)	10 (U)	15 (U)	13 (U)	12 (U)	10 (U)	14 (U)	18 (U)	27 (0.4)	13 (U)	18 (U)	17 (U)	11 (U)	10 (U)	18 (U)	17 (U)	22 (0.3)	57
Metro 12–17		113 (0.6)	134 (0.7)	167 (0.8)	166 (0.8)	193 (0.9)	235 (1.1)	221 (1.0)	248 (1.1)	282 (1.3)	244 (1.1)	232 (1.1)	238 (1.1)	232 (1.1)	161 (0.8)	165 (0.8)	175 (0.8)	192 (0.9)	70
Nonmetro 12–17		15 (U)	27 (0.7)	39 (1.0)	41 (1.0)	43 (1.1)	52 (1.3)	50 (1.2)	63 (1.6)	57 (1.5)	63 (1.6)	46 (1.2)	53 (1.4)	34 (0.9)	49 (1.4)	41 (1.1)	30 (0.8)	40 (1.1)	167
Metro 18–25		1,210 (4.7)	1,370 (5.2)	1,526 (5.7)	1,910 (7.0)	2,258 (8.1)	2,482 (8.7)	2,646 (9.2)	3,194 (11.0)	3,284 (11.2)	3,297 (11.1)	3,168 (10.6)	3,424 (11.4)	3,708 (12.2)	3,521 (11.5)	3,753 (12.1)	3,977 (12.8)	4,533 (14.6)	275
Nonmetro 18–25		114 (2.5)	156 (3.4)	232 (4.9)	306 (6.4)	395 (8.1)	473 (9.6)	519 (10.5)	636 (13.0)	636 (13.1)	544 (11.2)	552 (11.4)	557 (11.6)	564 (11.6)	508 (10.4)	516 (10.5)	513 (10.4)	583 (11.9)	411
Metro 26–34		2,749 (8.7)	2,625 (8.4)	2,787 (9.0)	3,314 (10.7)	3,477 (11.3)	3,556 (11.6)	4,117 (13.5)	4,778 (15.6)	5,025 (16.3)	5,083 (16.2)	5,383 (16.9)	5,777 (18.0)	6,507 (19.9)	6,657 (20.0)	7,045 (20.9)	7,927 (23.2)	9,377 (27.1)	241
Nonmetro 26–34		292 (5.9)	320 (6.5)	387 (8.1)	510 (10.8)	641 (13.7)	677 (14.6)	736 (16.0)	926 (20.2)	981 (21.2)	1,034 (22.1)	1,037 (21.9)	1,138 (23.9)	1,195 (24.8)	1,159 (24.1)	1,114 (23.1)	1,260 (26.2)	1,428 (29.7)	389
Metro ≥35		10,990 (9.4)	11,231 (9.5)	12,394 (10.3)	15,056 (12.3)	16,265 (13.1)	17,049 (13.5)	18,464 (14.4)	21,009 (16.1)	21,938 (16.6)	22,156 (16.6)	22,409 (16.5)	22,812 (16.7)	24,332 (17.5)	24,848 (17.7)	26,515 (18.7)	28,124 (19.5)	30,868 (21.1)	181
Nonmetro ≥35		1,293 (5.5)	1,494 (6.2)	1,784 (7.4)	2,135 (8.8)	2,431 (9.9)	2,817 (11.4)	2,974 (11.9)	3,461 (13.7)	3,704 (14.5)	3,934 (15.3)	4,084 (15.8)	4,241 (16.3)	4,683 (18.0)	4,512 (17.3)	4,745 (18.2)	4,963 (19.0)	5,272 (20.1)	308

**Abbreviations:** AI/AN = American Indian/Alaska Native; A/PI = Asian/Pacific Islander; U = unreliable (i.e., death count <20).

* Rates per 100,000 persons are adjusted to the 2000 U.S. standard population by the direct method. The NCHS six classification levels for counties are 1) large central metropolitan: part of a metropolitan statistical area with ≥1 million population and covers a principal city; 2) large fringe metropolitan: part of a metropolitan statistical area with ≥1 million population but does not cover a principal city; 3) medium metropolitan: part of a metropolitan statistical area with ≥250,000 but <1 million population; 4) small metropolitan: part of a metropolitan statistical area with <250,000 population; 5) micropolitan (nonmetropolitan): part of a micropolitan statistical area (has an urban cluster of ≥10,000 but <50,000 population); and 6) noncore (nonmetropolitan): not part of a metropolitan or micropolitan statistical area. For a dichotomous measure, metropolitan combines categories 1–4 and nonmetropolitan combines 5 and 6.

^†^ Race not determined in 3,041 deaths.

^§^ Hispanic origin can be of any race; other race categories exclude Hispanic origin.

**^¶^** Subnational data representing <10 (0–9) persons are suppressed.

** Age not determined in 133 deaths.

The age-adjusted drug overdose death rate for females was higher in metropolitan areas during 1999–2003 and higher in nonmetropolitan areas thereafter (Table 1). The difference in rates (per 100,000 population) between areas was greatest in 2015 (metropolitan: 11.5; nonmetropolitan: 14.3). The percentage change in the number of drug overdose deaths for females increased nearly 350% in nonmetropolitan areas during 1999–2015 (the percentage change in the age-adjusted death rate per 100,000 persons was 360%). The drug overdose death rate for males was higher in metropolitan areas in all years except 2010 and 2011 with the largest difference between metropolitan and nonmetropolitan rates occurring in 1999 (metropolitan: 8.8; nonmetropolitan: 4.8).

During 1999–2015, age-adjusted drug overdose death rates varied by race/ethnicity and metropolitan/nonmetropolitan area. American Indians/Alaska Natives had the highest drug overdose death rates in 2015 (metropolitan: 22.1; nonmetropolitan: 19.8) and the largest percentage change increase in the number of deaths over time (nonmetropolitan: 519%) (Table 2). During 1999–2001, black decedents in metropolitan areas had the highest death rates compared to other race/ethnicity categories; the rate for white decedents in metropolitan areas was highest in 2002 and 2007, and the rate was highest in remaining years for American Indians/Alaska Natives residing in metropolitan areas. Rates for white decedents (metropolitan: 21.4) were similar to American Indians/Alaska Natives in 2015. Drug overdose death rates were lowest among Asians/Pacific Islanders in all years.

Nonmetropolitan unintentional age-adjusted overdose death rates changed from 2.5 in 1999 to 14.2 in 2015 (Table 2). Metropolitan unintentional overdose death rates changed from 4.3 in 1999 to 13.8 in 2015. Rates of suicide overdose deaths were similar in metropolitan (1.5) and nonmetropolitan (1.6) areas in 2015.

All age group categories showed increases in drug overdose deaths from 1999 to 2015 (Table 2). The percentage change increase in age-specific drug overdose deaths from 1999 to 2015 was higher for nonmetropolitan areas than metropolitan areas for persons aged ≥12 years, with the largest increase in drug overdose deaths (411%) among those aged 18–25 years. Nonmetropolitan drug overdose death rates in 2015 were higher than metropolitan rates for those aged 26–34 years.

More age-adjusted drug overdose deaths occurred in a home versus in a medical facility or other location in each year for both metropolitan and nonmetropolitan areas (Table 3). The distribution changed over time, however, and the percentage of deaths that occurred in a home increased from 1999 to 2015 in both metropolitan and nonmetropolitan areas (1999 metropolitan: 45.2% versus 2015 metropolitan: 51.7%; 1999 nonmetropolitan: 43.3% versus 2015 nonmetropolitan: 53.5%).

**TABLE 3:** Number and age-adjusted rate* per 100,000 persons for drug overdose deaths, by place of death for metropolitan and nonmetropolitan counties of residence — National Vital Statistics System, United States, 1999–2015

Place of death	1999	2000	2001	2002	2003	2004	2005	2006	2007	2008	2009	2010	2011	2012	2013	2014	2015
	No. (%)																
**Metro**																	
Medical facility	5,148 (34.0)	5,291 (34.3)	5,683 (33.6)	6,600 (32.2)	6,865 (30.8)	7,050 (30.1)	7,492 (29.4)	8,758 (29.9)	8,668 (28.3)	8,235 (26.7)	8,195 (26.2)	8,784 (27.2)	9,707 (27.9)	9,438 (26.8)	10,059 (26.8)	10,561 (26.2)	12,006 (26.6)
Home	6,839 (45.2)	6,964 (45.2)	7,872 (46.5)	9,776 (47.7)	11,055 (49.7)	11,896 (50.9)	13,000 (50.9)	14,865 (50.7)	15,973 (52.2)	16,345 (53.0)	16,628 (53.2)	17,588 (54.4)	18,683 (53.6)	19,219 (54.5)	20,078 (53.5)	21,364 (53.0)	23,275 (51.7)
Other^†^	3,133 (20.7)	3,153 (20.5)	3,382 (20.0)	4,136 (20.2)	4,343 (19.5)	4,448 (19.0)	5,028 (19.7)	5,698 (19.4)	5,963 (19.5)	6,282 (20.4)	6,443 (20.6)	5,951 (18.4)	6,463 (18.5)	6,607 (18.7)	7,410 (19.7)	8,347 (20.7)	9,778 (21.7)
**Nonmetro**																	
Medical facility	691 (40.0)	743 (37.0)	914 (37.2)`	1,073 (35.7)	1,292 (36.7)	1,326 (32.9)	1,450 (33.8)	1,610 (31.5)	1,613 (29.8)	1,605 (28.7)	1,621 (28.3)	1,736 (28.9)	1,849 (28.5)	1,794 (28.8)	1,828 (28.4)	1,947 (28.7)	2,058 (28.0)
Home	748 (43.3)	951 (47.4)	1,182 (48.1)	1,476 (49.1)	1,725 (49.0)	2,099 (52.1)	2,192 (51.1)	2,655 (52.0)	2,904 (53.7)	2,994 (53.6)	3,006 (52.4)	3,242 (54.0)	3,510 (54.1)	3,364 (53.9)	3,461 (53.8)	3,638 (53.6)	3,931 (53.5)
Other	290 (16.8)	313 (15.6)	361 (14.7)	457 (15.2)	505 (14.3)	605 (15.0)	651 (15.2)	839 (16.4)	889 (16.4)	989 (17.7)	1,111 (19.4)	1,028 (17.1)	1,128 (17.4)	1,080 (17.3)	1,146 (17.8)	1,198 (17.7)	1,356 (18.5)

* Rates per 100,000 persons are adjusted to the 2000 U.S. standard population by the direct method.

^†^ Includes nursing home, hospice care, other, and unknown locations

**FIGURE 2:**
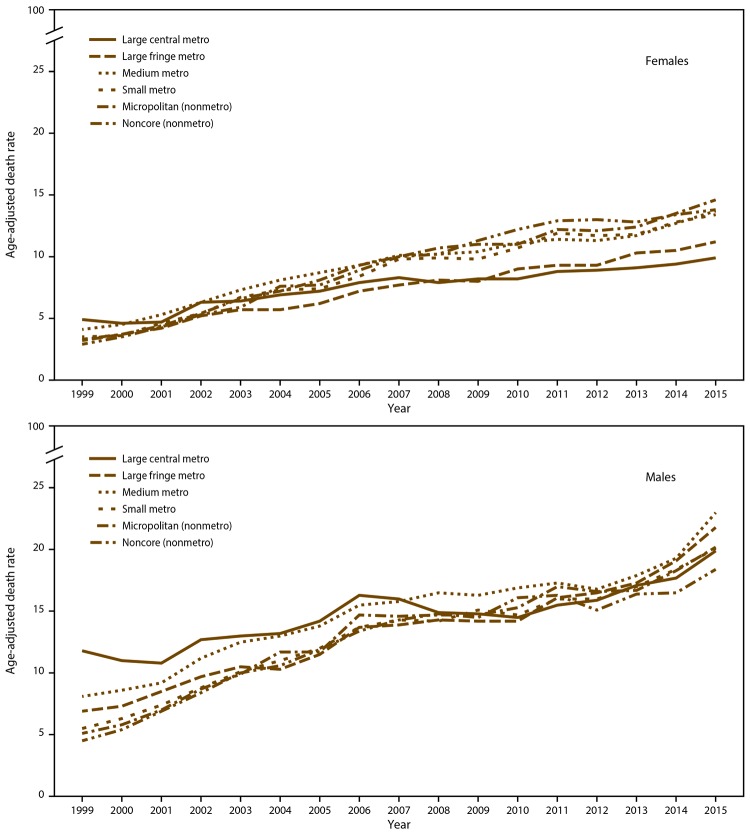
Age-adjusted rates* for drug overdose deaths, by sex and residential area^†^ — National Vital Statistics System, United States, 1999–2015 * Death rates per 100,000 persons were adjusted to the 2000 U.S. standard population by the direct method. ^†^ Uses the National Center for Health Statistics six classification levels for counties: 1) large central metropolitan: part of a metropolitan statistical area with ≥1 million population and covers a principal city; 2) large fringe metropolitan: part of a metropolitan statistical area with ≥1 million population but does not cover a principal city; 3) medium metropolitan: part of a metropolitan statistical area with ≥250,000 but <1 million population; 4) small metropolitan: part of a metropolitan statistical area with <250,000 population; 5) micropolitan (nonmetropolitan): part of a micropolitan statistical area (has an urban cluster of ≥10,000 but <50,000 population); and 6) non-core (nonmetropolitan): not part of a metropolitan or micropolitan statistical area.

The increasing trends for males and females in age-adjusted drug overdose death rates varied by the six urban levels (Figure 2). Drug overdose death rates had a greater range by level of urban status for males in 1999 (noncore = 4.5 to large central metropolitan = 11.8) than in 2015 (noncore = 18.4 to medium metropolitan = 23.0). Converse patterns were observed for females (1999 range: noncore = 2.9 large central metropolitan = 4.9; 2015 range: large central metropolitan = 9.9 to micropolitan nonmetropolitan = 14.6). At the beginning of the study period, death rates were higher in metropolitan areas than in nonmetropolitan areas, but the rates converged over time.

## Discussion

This report presents an overview of illicit drug use, illicit drug use disorders, and drug overdose deaths for metropolitan and nonmetropolitan areas in the United States. The findings of this study indicate that trends varied. On the one hand, the decline in illicit drug use by youth and the lower prevalence of illicit drug use disorders are encouraging signs. On the other hand, the increasing rate of drug overdose deaths in rural areas, which surpassed rates in urban areas, is cause for concern. Regardless of metropolitan/nonmetropolitan area, illicit drug use among youth generally declined over a 10-year period, but increased substantially in other age groups (aged 26–34 and ≥35 years). Declines in prescription opioid use disorders have also been demonstrated among persons aged 12–17 years (*15*). The percentage change increase between 1999 and 2015 in overdose deaths among nonmetropolitan residents is concerning and carries across sex, race, and intent. The type of drug involved in overdose deaths varies by metropolitan/nonmetropolitan area with rates of heroin and cocaine related overdose deaths being higher in more urban areas (*7*); however, monitoring trends in use and drugs involved in overdoses is critical as they have changed rapidly in recent years (*16*). Although past-month use of illicit drugs was lower in nonmetropolitan areas compared to metropolitan areas, the prevalence of drug use disorders among people reporting past-year illicit drug use in nonmetropolitan areas was similar to that in metropolitan areas. Studies have found that persons with substance use disorders are at higher risk for drug use-related morbidity and mortality (*17*). Further, given research indicating that nonmetropolitan areas have less access to substance abuse treatment services (*18*) and other risk reduction strategies (*19*), the similar prevalence of drug use disorders in this study underscores the importance of scaling up these critical interventions in nonmetropolitan areas.

Because of the involvement of prescription opioids in the current epidemic (*20*), monitoring prescribing levels along with understanding the local illicit drug trade is important for prevention efforts. Recent studies suggest that a leveling off and decline has occurred in opioid prescribing rates since 2012 and in high-dose prescribing rates since 2009 (*7*). However, overall opioid prescribing remained high in 2015 and the amounts prescribed varied by level of urbanization (*21*). Reducing the number of persons initially exposed to prescription opioids might reduce the illicit use of opioids, the subsequent risk of addiction, and the use of illicit drugs (*22*).

Interventions for drug overdoses, such as naloxone administration, rescue breathing, or calling 911, are most useful when someone is present to administer them. In both metropolitan and nonmetropolitan areas, the majority of overdose deaths occurred in a home, and rescue care could fall to friends or relatives who might lack knowledge about naloxone administration and follow-up care. In private locations, such as homes, bystanders might not know to call for emergency services after giving naloxone (*23*). Further, naloxone is less often administered by emergency medical technicians-basics (persons trained to provide basic-level life support), who are more common in rural areas (*24*) than paramedics (who can provide advanced life support care). Legislation designed to improve access to naloxone by laypersons is now present in all 50 states and the District of Columbia (*25*).

Although prevalence rates of drug use in rural areas are lower in this report, the consequences of use appear to be higher. For example, an investigation of HIV infections linked to the injection of prescription opioids found that the majority of the counties at high risk for rapid dissemination of hepatitis C virus or HIV were in rural areas (*26*). Access to substance abuse treatment services is more limited in rural areas (*27*) and strengthening the health care delivery system while improving the integration of primary, specialty, and substance abuse services can provide the nexus of care needed to reduce drug misuse (*28*–*31*). Specific interventions to address overdose deaths in rural areas have been discussed previously (*25*) and might include actions such as expanding the types of emergency medical service providers that can administer naloxone to reverse a drug overdose. In addition, the following resources can assist providers and communities in efforts to reduce misuse and overdose:

- CDC’s rural health website (https://www.cdc.gov/ruralhealth/) and the Guideline for Prescribing Opioids for Chronic Pain resource page (https://www.cdc.gov/drugoverdose/prescribing/resources.html;
- the Substance Abuse and Mental Health Service Administration’s Opioid Overdose Toolkit (https://store.samhsa.gov/shin/content//SMA14-4742/Overdose_Toolkit.pdf) or the Bureau of Justice Assistance Law Enforcement Naloxone Toolkit website (https://www.bjatraining.org/tools/naloxone/Naloxone-Background);
- the U.S. Department of Health and Human Services resource of tools and information for families, health care providers, law enforcement, and other stakeholders about prescription drug abuse and heroin use prevention, treatment, and response (https://www.hhs.gov/opioids); and
- the Rural Health Information Hub’s toolkit, which provides evidence-based examples, promising models, program best practices, and resources that can be used to implement substance abuse prevention and treatment programs (https://www.ruralhealthinfo.org/community-health/substance-abuse).

## Limitations

The findings in this report are subject to at least six limitations. First, NSDUH is a self-reported survey and is subject to recall bias and social desirability; however, good validity and reliability have been found for substance use measured by NSDUH (*32*). Second, NSDUH excluded persons who were homeless and not living in shelters or persons residing in institutions, which could lead to underestimates in drug use and drug use disorders. Some areas might change in their urbanization categorization over time; however, few would have changed from the larger grouping of metropolitan to nonmetropolitan (*14*). In addition, the 2013 NCHS county-based classification scheme was used for the entire time period for parsimony. Although metropolitan/nonmetropolitan areas were not directly comparable between the two data systems, results demonstrate that it is important to consider the rural to urban continuum of population density for drug use and overdose. Third, overdose deaths are likely underestimated because lengthy investigations are often required including toxicology assessments and this sometimes results in a death being categorized as “pending manner and cause of death” at the time mortality files are closed and shared with NCHS. It was not possible to analyze this underestimation by urban level directly. Fourth, because of the misclassification of race/ethnicity of decedents on death certificates, the actual numbers of deaths for certain racial/ethnic populations (e.g., American Indians/Alaska Natives and Hispanics) might be underestimated by up to 35% (*33*). Fifth, because information on intent might be coded in the mortality file even though the case could still be under investigation, it might be difficult to determine whether a death should be certified as suicide or unintentional (*34*). Finally, the time periods were different between the two data systems (NVSS-M: 1999–2015; NSDUH: 2003–2014), so the duration of change over time varied.

## Conclusion

Drug use and subsequent overdoses continue to be a critical and complicated public health challenge. Variations and trends in drug overdose death rates by urban status differed by sex and race/ethnicity and trends among these subgroups indicate that certain groups are more profoundly affected by the epidemic than others. The decline in illicit drug use by youth and the lower prevalence of illicit drug use disorders in rural areas during 2012–2014 are encouraging signs. However, the rising death rate of drug overdoses in rural areas, which surpassed rates in urban areas, along with persistent limited access to substance abuse treatment services in rural areas is cause for concern. Educating opioid prescribers on the CDC Guideline for Prescribing Opioids for Chronic Pain (*35*) and better access to evidence-based substance abuse treatment, including medication-assisted treatment for opioid addiction, are critical steps that can be taken in communities heavily impacted by substance abuse.
