# *Erratum*: Vol. 66, No. SS-19

**DOI:** 10.15585/mmwr.mm6645a6

**Published:** 2017-11-17

In the Surveillance Summary “Illicit Drug Use, Illicit Drug Use Disorders, and Drug Overdose Deaths in Metropolitan and Nonmetropolitan Areas — United States,” on page 8, the title of Table 2 should have read “TABLE 2. Number and age-adjusted rate per 100,000 persons for drug overdose deaths, by sex, race/ethnicity, intent of death, and age group, for metropolitan and nonmetropolitan counties of residence — National Vital Statistics System, United States, 1999–2015.*”

